# Effects of the COVID-19 lockdown on mental health in a UK student sample

**DOI:** 10.1186/s40359-022-00732-9

**Published:** 2022-05-07

**Authors:** J. C. Catling, A. Bayley, Z. Begum, C. Wardzinski, A. Wood

**Affiliations:** School of Psychology, University of Birmingham, 52 Pritchatts Road, Edgbaston, Birmingham, UK

**Keywords:** COVID, Mental Health, Depression, Anxiety, Smartphone

## Abstract

**Background:**

The COVID-19 pandemic and the resulting restrictions placed upon society have had a profound impact on both physical and mental health, particularly for young people.

**Aims:**

The current study assesses the impact of COVID-19 on student mental health.

**Method:**

Four hundred and thirty four first year Undergraduate students completed a battery of self-report questionnaires (PHQ-P, GAD-7 and SAS-SV) to assess for depression, anxiety and mobile phone addiction respectively with data being collected over a 2 year period. The data from each year was compared (216 and 218 students respectively).

**Results:**

A MANOVA revealed that COVID-19 had a significant impact on self-reported levels of depression, anxiety and smartphone addiction—which all significantly increased from the 2020 to the 2021 group. The percentage of students who had a score which warranted a classification of clinical depression increased from 30 to 44%, and for anxiety increased from 22 to 27%—those students who showed a comorbidity across the two rose from 12 to 21%. Smartphone addiction levels rose from 39 to 50%. Correlational analysis showed a significant relationship between Smartphone usage and depression and anxiety.

**Conclusions:**

This research suggests that COVID-19 has had a major impact upon student mental health, and smartphone addiction. The importance of identifying predictive factors of depression and anxiety is emphasised, and suggestions for intervention are discussed.

## Introduction

The coronavirus disease 2019 (COVID-19) pandemic has had a profound impact on the world’s population. Furthermore, the consequent ‘lockdowns’ and social restrictions have had an unknown impact on the physical and mental health of society particularly for University students. Even with the partial lifting of restrictions at the beginning of the 20/21 academic year, the majority of students were taught in the main online and had none of the usual social interactions associated with University life—with many returning to their family home at some point during the academic year. We know little about how the COVID-19 pandemic and ensuing restrictions have impacted mental health, in particularly in our young people in Higher Education (H.E.).

As a result of COVID-19, Marshall et al. [1] have calculated a worsening of general mental health by 8.1%, particularly affecting young adults and women. Salari et al. [2] identified in a meta-analysis of over 9000 people that prevalence rates for depression, anxiety and stress in the time of the pandemic were around 30%. Stress may be mediating this increase—Montano and Acebes [3] identified that COVID-related stress did indeed predict increased depression and anxiety. In an online survey of 2,000 participants, it was found that specifically the social isolation of the COVID-19 pandemic created feelings of anxiety and depression, Rahman [4]. This impacted how students were able to engage with their learning, as some students refused to join online classes or were unwilling to participate in online activities due to the negative impact on their poor mental health. Elhai et al. [5] also identified that COVID-related anxiety positively correlated with smartphone use. However, there are few empirical comparisons of these factors pre- and post-COVID-19, from comparable samples, to provide quantitative evidence for COVID-19’s effect on Mental health. Examples that do measure the impact on mental health of the early lockdown come from Huckins et al. [6], who in a 2 year longitudinal study of U.S. students, that ended in Easter 2020, found that individuals in the latter points of the study (where COVID-19 was considered a global pandemic); were more sedentary and reported increased anxiety and depression symptoms relative to previous academic terms. They found that phone usage, number of locations visited, depression and anxiety were all strongly associated with increased amount of COVID-19–related news. Similarly, Kaparounaki et al. [7], in a survey of 1000 Greek University students mental health, found that there was a dramatic increase in scores for anxiety (42.5%), 74.3% for depression, and a 63.3% increase in total suicidal thoughts. Quantity of sleep increased in 66.3% but quality worsened in 43.0%. Quality of life worsened in 57.0% (same in 27.9%). Furthermore, Evans et al. [8] –found that early Covid restrictions had significantly increased levels of depression and surprisingly reduced alcohol consumption in a student population (the latter may be due to fewer opportunities for social interaction). Conversely, Fancourt et al.’s [9] longitudinal study suggested that depressive and anxious symptoms will decline as individuals acclimatise to the lockdown, thus comparing data from pre- and post-lockdown is of great interest. Li et al. [10] in a large-scale, longitudinal, population-based survey conducted among college students in China, assessed the rates of three mental health problems (acute stress, anxiety, and depressive symptoms), at two time points; at the initial onset of the pandemic and, during the later 2nd wave, the COVID-19 remission stage in China. They found that while the prevalence of acute stress symptoms decreased, conversely the rates of depression and anxiety increased over the course of the epidemic.

We know that students in H.E. can experience a range of added risk factors, such as academic, financial and social pressures [11]. These pressures can impact on a student’s academic progression through higher education and can also lead to mental health problems including, specifically most commonly depression and anxiety [12]. Wyatt and Oswalt [13] focused on the impacts of stress on mental health issues amongst university students compared to graduates. They found that undergraduates reported significantly higher rates of poor mental health, which led to a negative impact upon their academic performance. In particular they found that the transition to university life could have a huge impact on student’s mental health. Therefore, it would seem important to research the specific predictors that contribute to the onset of anxiety and depression in university students, as this may be of importance for developing preventative measures and reducing the negative impacts of poor mental health. One possible predictor could be the relatively recent introduction of Smartphones to the younger population.

Over the past decade Smartphone-use has become an essential part of young people’s lives, for many it can be a positive addition to their lives, increasing connectivity and allowing people to share common experiences. This can create support through virtual environments, and potentially have a positive impact on Mental Health [14]. However, when smartphone-use becomes excessive, it may consequently increase mental health problems. For instance, self-reports from a study by Elhai et al. [5] (with Chinese students) found that problematic smartphone-use amongst undergraduate student’s led to an increase in depression and anxiety symptoms (see also Boumosleh & Jaalouk [15]; Grant et al. [16]). These findings were supported by a meta-analysis of 41 studies by Sohn et al. [17], who found strong evidence that problematic smartphone use resulted in an increased risk for both depression and anxiety in young people. Furthermore, Elhai et al. [18] in a systematic review found that depression, and anxiety were consistently related to problematic smartphone usage with a small to medium effect size.

Problematic smartphone-use has been explained by the ‘excessive reassurance pathway’ [19]. This states that smartphone-use becomes problematic when individuals feel the need to gain reassurance from others. This derives from a lack of emotional stability in ‘real’ life, thus causing individuals to strive to maintain relationships online. Consequently, ‘dependent users’ more likely display symptoms of depression and anxiety. Supporting qualitative research indicates that problematic smartphone-use is linked to the excessive reassurance pathway, where students experienced fear of missing out (FOMO) when they were not using their devices [20]. While smartphone addiction is not a formal definition used by the DSM, it is known that young people have become more and more reliant on their smartphones. Notably, reports show that 63% of 18–24-year-olds cannot go more than 2 days without a smartphone, compared to 54% across all age groups [21], suggesting an addictive nature to their use in young people. In a sample of UK undergraduate students, smartphone addiction was present in 39% of participants [22].

Following from this, the current study assesses the impact of the Covid-19 pandemic on student Mental health and Mobile phone use, and the relationship between these variables. Specifically we predict a significant increase in levels of depression, anxiety and mobile phone use (Post-COVID) and also a significant positive association between the 3 variables.

- Research Question 1: Has there been a significant increase in levels of depression, anxiety and mobile phone use due to the impact of COVID-19?
- Research Question 2: Has there been a significant increase in the proportion of students that are clinically classified with depression, anxiety or mobile phone addiction due to the impact of COVID-19?
- Research Question 3: Is there a significant association between depression, anxiety and mobile phone use?

## Method

### Participants

434 first-year undergraduate students aged 17–35 were recruited via a research participation scheme for which they received credits for completing the questionnaire. The 2020 group comprised 216 students (M = 18.5 years, SD = 0.894, 83.3% female), the 2021 group comprised 218 students (M = 18.8 years, SD = 1.49, 86.2% female).

## Measures

*Patient Health Questionnaire* (PHQ-9; Kroenke et al. [23]) was used as a self report measure of depression severity. The questionnaire focuses on diagnostic criteria for depression (DSM-IV), assessing severity via nine questions on a scale from experiencing a problem ‘not at all’ (0) to ‘nearly every day’ (3) over the last 2 weeks (e.g. ‘Feeling down, depressed or hopeless’). Higher scores represent higher depression severity, the highest possible score is 27. Internal consistency was reviewed with a Cronbach’s *α* coefficient of 0.86, with good test retest reliability. Beard et al. [24] identified good convergent and discriminant validity in a psychiatric sample. Manea et al. [25] identified an optimal cut-off score of 10 when diagnosing (moderate to severe) depression with the PHQ-9 with 88% sensitivity and 88% specificity.

*Generalised Anxiety Disorder-7* (GAD-7; Spitzer et al. [26]) was used as a self-report measure of anxiety. Seven symptoms of anxiety based on diagnostic criteria (DSM-IV) are measured, from the problem bothering an individual ‘not at all’ (0) to ‘nearly every day’ (3) over the last 2 weeks (e.g. ‘Trouble relaxing’). Higher scores represent higher anxiety severity, the highest possible score is 21. Cronbach’s *α* coefficient for internal consistency was measured at 0.92, and test–retest reliability correlated at 0.83, and good criterion and procedural validity was shown. Spitzer et al. [26] identified a cut-off score of 10 when diagnosing (moderate to severe) anxiety with the GAD-7 with 89% sensitivity and 82% specificity.

*Smartphone Addiction Scale Short-Version* (SAS-SV; Kwon et al. [27]) was used as a self-report measure of SA. The questionnaire contains 10 statements which are measured on a scale from 1 to 6 (strongly disagree to strongly agree; e.g. ‘Using my smartphone longer than I had intended’), with a highly reliable Cronbach’s *α* coefficient of 0.91. Higher scores represent higher SA, the highest possible score is 60. The cut-off value for considering an individual ‘addicted’ to their smartphone was 31 in males, and 33 in females. Andrade et al., [28] showed that the questionnaire had good predictive and convergent validity in adolescents. Kwon et al. [27] suggested a cut-off value of 33 to signify clinical addiction.

### Procedure

Participants volunteered to partake in the study via the University’s website. Participants completed all sections of the questionnaire and submitted responses via Google Forms; completion took ~ 30 min. Data from the 2020 group were collected from Oct 2019 to end of Jan 2020 (Pre-Covid), data from the 2021 group were collected from Oct 2020 to end of Jan 2021 (Within-Covid). Results were collated and analysed.

### Ethical considerations

Ethical permission was obtained from the University of Birmingham’s Ethics committee. Participants consented to participate and were informed of their right to withdraw data from analysis prior to a given date. Student ID numbers were used, maintaining confidentiality. A variety of mental health service resources were highlighted should participants have any concerns relating to the content of the questionnaires.

All experimental protocols were approved by the University of Birmingham’s ethics committee.

All methods were carried out in accordance with relevant guidelines and regulations.

Informed written consent was obtained from all participants.

## Results

Statistical comparisons between the 2020 and 2021 groups for Gender and Age were undertaken- for Age an independent t-test showed no significant difference between the groups (T = 0.652, p > 0.05); for gender a Pearsons Chi-squared analysis showed no significant difference between the groups (χ^2^(1) = 2.4, p > 0.05).

### Results for Research Question 1

Descriptive statistics including means, standard deviations and ranges were calculated for both groups. Depression, anxiety and SA increased compared to the previous year (see Table 1). To check for significant effects of COVID-19, a MANOVA was conducted, with groups as the between-subjects factor.

**Table 1:** Descriptive statistics of both groups of data with the means, standard deviations and ranges, including the change in mean from 2020 to 2021

Variable	2020 Group (*N* = 216)		2021 Group (*N* = 218)	
	Mean (*SD*)	Range	Mean (*SD*)	Range
Depression**	8.1 (4.1)	19 (0–19)	9.6 (5.8)	27 (0–27)
Anxiety*	6.1 (4.1)	18 (0–18)	7.1 (5.1)	21 (0–21)
Smartphone Addiction**	30.3 (8.5)	43 (10–53)	32.6 (9.1)	45 (10–55)

*Significant change at *p* < 0.05, **Significant change at *p* < 0.01

The MANOVA revealed a significant main effect of Group for depression (*F*(1, 432) = 8.99, p = 0.003, η^2^ = 0.020). The 2020 Group (*M* = 8.1, *SD* = 4.1) experienced significantly lower levels of depression than the 2021 cohort (*M* = 9.6, *SD* = 5.8). A significant main effect of cohort was found for anxiety (*F*(1, 432) = 4.53, *p* = 0.034, η^2^ = 0.010). The 2020 Group (*M* = 6.1, *SD* = 4.1) experienced significantly lower levels of anxiety than the 2021 group (*M* = 7.1, *SD* = 5.1). A significant main effect of Group was found for Smartphone use (*F*(1, 432) = 7.36, *p* = 0.007, η^2^ = 0.017). The 2020 group (*M* = 30.3, *SD* = 8.5) experienced significantly lower levels of Smartphone use than the 2021 group (*M* = 32.6, *SD* = 9.1).

### Results for Research Question 2

**Fig. 1:**
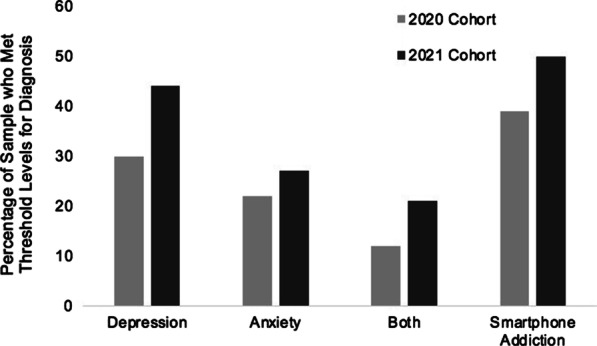
Proportions of 2020 and 2021 groups who met clinical thresholds for depression, anxiety, both depression and anxiety, and smartphone addiction

Clinical significance was explored by calculating the proportion of students who met clinical thresholds for moderate to severe depression or anxiety. Depression levels increased from 30 to 44%, anxiety from 22 to 27%, and the chance of reporting both rose from 12 to 21%. The proportion of Smartphone addiction scores meeting the criterion for clinical addiction rose from 39 to 50% (see Fig. 1). A series of Pearson Chi-square analyses was undertaken to identify whether there were significant differences in the proportions of clinical depression, anxiety and smart phone addiction levels between groups. Results of the Chi-square showed a significant difference in depression between groups (χ^2^(1) = 11.8, p < 0.001); no significant difference in anxiety between groups (χ^2^(1) = 1.1, p = 0.288); a significant difference in smart phone addiction between groups (χ^2^(1) = 5.9, p < 0.05).

### Results for Research Question 3

In a second phase of analysis, Pearson correlations were conducted for both groups (see Tables 2 and 3). These revealed similar results—as expected anxiety and depression were significantly positively correlated, however it was also found for both groups that levels of Smartphone use were also significantly positively correlated with both depression and anxiety, as smartphone usage went up so did levels of depression and anxiety.

**Table 2:** Correlations between all variables, 2020 Group (N = 216)

Variable	1	2	3
1. Depression	–	.647**	.255**
2. Anxiety	.647**	–	.210**
3. Smartphone Addiction	.255**	.210**	–

**Significant change at *p* < 0.01

**Table 3:** Correlations between all variables, 2021 Group (N = 218)

Variable	1	2	3
1. Depression	–	.728**	.249**
2. Anxiety	.728**	–	.249**
3. Smartphone Addiction	.249**	.249**	–

**Significant change at *p* < 0.01

## Discussion

The current study assessed the effects of COVID-19 on student mental health. Results showed that COVID-19 had a significant impact upon depression, anxiety and Smartphone use/addiction. The proportion of the 2021 group who met clinical thresholds for depression was significantly higher, reaching 44%. Furthermore, worryingly, half of our COVID group were classified as being addicted to their smartphone. We also found significant correlation between mental health problems and smartphone usage, showing a clear association between increases in smartphone use and increases in depression and anxiety.

The findings of the current study corroborated both Fried et al. [29] and Evans et al. [8] findings that depressive symptoms have increased during COVID-19. Of particular interest was the significant increase in moderate-severe depression levels between groups: an increase of 14.0%. There are no studies to date which have examined differences between groups of UK students cross-sectionally before and during the full pandemic. However, studies such as Elmer et al. [30] do report similar findings in a Swiss sample, showing increased depression and anxiety in an ‘early’ COVID-19 cohort group compared cross-sectionally with pre-COVID-19 students. Furthermore, although Fried et al., [29] observed decreases in anxiety, loneliness, and COVID-19-related concerns, during the transition to a partial Covid ‘lockdown’ in the Netherlands, conversely, they also saw other mental health variables, such as stress levels, remained stable, or in the case of depressive symptoms, increase. Their analysis identified potential vicious cycles between mental health variables and being alone, which predicted concerns about COVID-19 and was followed by further mental health problems.

This converging evidence highlights the clear negative impact of the pandemic on mental health and should trigger future research to investigate this further. There was also as expected a strong significant positive correlation between depression and anxiety, indicating a high level of comorbidity for these conditions. Importantly, the prevalence rates for depression and anxiety in the 2020 group was already higher than in the general population showing that students on average have poorer mental health in comparison to the adult population. This is in line with the findings of Thorley [12] who found that in England, 19% of 16–24-year-olds experience a mental health condition, Among this age group, 28% of women experience mental health problems, compared to 10% of men. The number of students who disclose a mental health condition to their university has also increased dramatically in the past 10 years, increasing almost fivefold.

The results from the current study support Ithnain et al.’s. [31] findings—that for students in Malaysia that there was a statistically significant positive relationship between smartphone addiction with anxiety and depression, and that smartphone addiction was also found as a predictor of both anxiety and depression. Similarly, Elhai et al. [18] found that depression and anxiety were related to problematic smartphone use. The significant increase in problematic smartphone use between groups also aligns with reports of smartphone use increasing during the pandemic (e.g., Zuckerman [32]) and could be due to the lack of opportunities for face-to-face communication. However, one limitation of measuring ‘smartphone use’ is that this term can cover a range of areas that may have variable effects on mental health. Indeed, evidence has found that social networking was the preferred activity for problematic smartphone users (Sohn et al. [22]), with gaining peer acceptance as the primary cause of smartphone overuse (Lee & Lee [33]). Therefore, it could be that social media use is driving the high prevalence rates of mental health problems, rather than smartphone use per se.

Our findings could be due to the students being part of a rigorous course which has become increasingly complex with the challenge of remote learning. Therefore, this sample may not generalize to the general population. Moreover, due to the anonymous nature of the study, students may have been more inclined to give truthful responses, which could further account for the high prevalence rates.

Limitations of the current study arise in terms of the over-representation of female participants in both groups. It is reported that females experience more mental health problems than males (e.g., Kuehner [34]; Li and Graham [35]), however this could be due to an increased likelihood of disclosure. Furthermore, there could be gender differences in smartphone use, such that male students with smartphone addiction are more likely to use gaming apps, whereas females prefer to use social media (Chen et al. [36]). Additionally, it should be noted that the current study is not based on longitudinal data, but on two cross-sectional studies, and hence any differences in the outcome measures between the groups must be tempered by the potential for underlying individual differences between the groups. Hence we suggest further research within this domain should focus on medium to long term longitudinal approaches.

The present findings should be of interest to universities, highlighting a critical time to intervene to safeguard their students. Thorley [12] has highlighted a 94% increase in demand for University counselling services, in some universities, up to 1 in 4 students are using, or waiting to use, counselling services. Broadly, we recommend that Universities should create campus environments to promote positive mental health, emphasising time away from smartphones. From a clinical perspective, our findings are particularly concerning, reflecting an urgent need for interventions. In addition to CBT and mindfulness interventions to improve mental health, research (e.g., Lan et al. [37]; Young [38]) also suggests that mindfulness and cognitive-behavioural techniques can be utilised as smartphone addiction interventions, to indirectly treat depression and anxiety in students. These findings should be used to demonstrate a growing public health concern that should inform public health policy to prevent a post-COVID-19 mental health crisis in UK universities.

## Data Availability

All data generated or analysed during this study are included in this published article.
